# Supporting couples’ perinatal sexual well-being: a feasibility study of the introductory modules of a novel prevention program (STORK)

**DOI:** 10.1186/s40814-026-01878-8

**Published:** 2026-07-21

**Authors:** Inês M. Tavares, Samantha J. Dawson, Erin T. Fitzpatrick, Greta E. Jang, Kyle R. Stephenson, Natalie O. Rosen

**Affiliations:** 1 Faculty of Psychology and Education, Hei-Lab–Digital Human-environment Interaction Lab, Lusófona University, Porto, Portugal; 2 Department of Psychology, University of British Columbia, Vancouver, BC Canada; 3 Department of Psychology and Neuroscience, Dalhousie University, Halifax, NS Canada; 4 School of Psychology, Xavier University, Cincinnati, OH USA; 5 Department of Obstetrics and Gynaecology, Dalhousie University, Halifax, NS Canada

**Keywords:** Pregnancy, Postpartum, Perinatal, Transition to parenthood, Psychoeducation, Internet intervention, Couple

## Abstract

**Background:**

Most new parents will experience changes to their sexual relationship during the perinatal period, yet no accessible prevention programs exist to support couples in navigating these changes. Our aim was to assess the feasibility of the introductory modules of an online couple-based program (STORK) designed to promote perinatal sexual well-being. The program is grounded in empirically established risk and protective factors associated with couples’ perinatal sexual well-being.

**Methods:**

We conducted a single-group feasibility study with eleven couples (22 individuals) expecting their first child (mean gestation 20 weeks). Couples completed the orientation and first STORK modules during a 2-h online session with a researcher. Participants then completed an online survey evaluating the program’s feasibility and content. Qualitative semi-structured interviews and online questionnaires assessed participants’ experiences with the platform, focusing on usability, acceptability, clarity, relevance, and overall satisfaction.

**Results:**

Participants reported high acceptability, rating both modules as clear and logical (*mean* = 5.6–5.9/7), enjoyable (*mean* = 4.7–6.1/7), helpful (*mean* = 5.0–5.7/7), and relevant (*mean* = 6.1/7). The platform was rated as easy to navigate (*mean* = 5.6/7) and highly inclusive (*mean* = 6.0–6.1/7). Most participants found the time commitment reasonable (95.5%), and 91% considered weekly completion with their partner feasible. Qualitative feedback highlighted usability, well-structured content, inclusivity, and support for personal and relationship reflection.

**Conclusions:**

The findings suggest that the STORK program is a feasible and acceptable resource to support couples’ perinatal sexual well-being, warranting further evaluation of its effectiveness. A pilot randomized controlled trial is warranted and may facilitate a shift toward couple-focused perinatal prevention programs. The STORK program has the potential to fill a critical gap in perinatal healthcare by providing an accessible, evidence-based prevention program to support couples navigating sexual changes during pregnancy and postpartum.

**Trial registration:**

This feasibility study was prospectively registered on June 17, 2025 (reg #2023-6719), and the registration can be found here: https://clinicaltrials.gov/study/NCT07036484

**Supplementary Information:**

The online version contains supplementary material available at https://doi.org/10.1186/s40814-026-01878-8.

## Key messages regarding feasibility

- What uncertainties existed regarding the feasibility?

It was unknown whether couples would be willing to engage with an online, couple-based program focused on sexual well-being during the perinatal period—a topic that is often under-discussed and sensitive. Uncertainties also included whether the platform would be considered usable and inclusive, whether the content would be seen as relevant and appropriate, and whether the required time commitment would be acceptable to expecting parents.

- What are the key feasibility findings?

The orientation and first content module of the STORK program were found to be highly feasible and acceptable to couples expecting their first child. Participants rated these introductory modules as clear, well-organized, and enjoyable. They reported that the platform was easy to navigate and visually appealing. The program was described as inclusive and representative, and it facilitated meaningful reflection and conversations between partners. Most participants felt the time commitment was reasonable and expressed strong interest in completing the remainder of the program.

- What are the implications of the feasibility findings for the design of the main study?

Findings support the feasibility of delivering the STORK program in its current format and content. No major revisions were required, and participant engagement and satisfaction suggest strong potential for uptake. A fully powered trial evaluating the program’s effectiveness is now warranted. Future work should explore outcomes across the full course of the intervention, including postpartum modules, and aim to recruit a more diverse sample to further assess accessibility and generalizability.

## Background

The perinatal period (i.e., pregnancy up until 12 months postpartum) is often an exciting yet challenging period for couples, particularly if couples are becoming parents for the first time. Pregnancy and the postpartum period involve biopsychosocial changes that impact couples’ sexuality [1, 2]. Consistent with a biopsychosocial framework, perinatal sexual well-being can be understood as resulting from interacting biological, psychological, relational, and social factors [1, 2]. This framework provides a basis for targeting modifiable factors, including knowledge of normative sexual changes, cognitive and affective responses to these changes, and couple communication and coping. Indeed, up to 88% of gestational parents (i.e., individuals who experience pregnancy) and up to 47% of non-gestational parents will experience sexual problems (e.g., sexual distress, low sexual desire, genito-pelvic pain) that may begin in pregnancy and persist up to 12-months postpartum and beyond [1]. Perinatal sexual difficulties are associated with a range of negative outcomes, such as poorer general health and quality of life, perinatal anxiety and depression, relational conflict and dissolution, and less sensitive parenting [3–5], as well as with increased health service utilization [5].

Despite the ubiquity of perinatal sexual changes, couples often report a lack of information about these issues. Only 7% to 29% of expectant parents report receiving any guidance from healthcare providers regarding potential sexual changes during the perinatal period [6–8], despite most wishing they had received such information [9]. When couples receive more information about their perinatal sexuality, both partners report higher sexual frequency, satisfaction, desire, and lower sexual distress in pregnancy and the postpartum period [9], highlighting the importance of providing such information during this period. Given that perinatal sexual well-being occurs within a dyadic context, interdependence theory further highlights the mutual influence of partners’ experiences, responses, and outcomes [1, 10]. This perspective supports targeting both gestational and non-gestational parents in efforts to promote couples’ sexual well-being during the perinatal period.

One key factor that may prevent perinatal sexual difficulties is having realistic expectations regarding perinatal sexual changes. Expectations play a key role in shaping sexual well-being because people evaluate their current situations by comparing them to prior expectancies [10, 11]. A recent study found that couples at 3 months postpartum felt less sexually satisfied when their postpartum sexual experience was worse than they expected (i.e., unmet expectations) and more sexually satisfied and less sexually distressed when their postpartum sexual experience was better or not as bad as they expected (i.e., exceeded expectations) [12]. Expectations can be shaped through having access to evidence-based information [10, 11] that helps people anticipate perinatal sexual changes, develop realistic expectations about these changes, as well as enhance their confidence in managing and reducing distress when such changes occur. This information might be especially relevant for first-time parents. Couples undergoing the transition to parenthood for the first time have no prior experience to guide their expectations, and evidence suggests they desire more realistic, evidence-based preparation for managing pregnancy and postpartum sexual changes than they receive [9].

### Existing interventions for perinatal sexual problems

Converging evidence suggests that psychoeducational approaches to perinatal sexuality are associated with improved sexual outcomes for expectant and new parents. For example, sexual education training early in the postpartum period has been linked with greater sexual function at 3-months postpartum compared to couples receiving standard perinatal care [13]. Furthermore, exposure to brief, online video content providing information about postpartum sexuality also appears to be beneficial for lowering concern about changes to perinatal sexual well-being and increasing parents’ confidence in their perceived ability to cope with and discuss sexual changes with a partner [14]. Importantly, couples-focused psychoeducational interventions related to pregnancy sexuality show that both parents report higher sexual function post-intervention compared to controls [15, 16]. While promising, these existing perinatal programs focused on expectant and new parents’ sexual health have been relatively limited such that they have mostly addressed sexual problems after they had already developed, neglected to include both members of the couple [13, 14], and typically required in-person attendance (e.g., [15, 16]), likely posing challenges for feasibility and accessibility during the busy time of new parenthood.

Reviews evaluating controlled interventions focusing on relationship (but not sexual) outcomes in the perinatal period indicate that the most successful programs, both in terms of feasibility and efficacy, are brief and easily accessible (e.g., online) [17–19]. Self-administered programs with some degree of professional support (e.g., contact via chat) have been particularly successful and cost-effective in improving both couple relationships and parenting in the perinatal period, and are positively endorsed by parents [17]. In line with these recommendations to meet the needs of expectant and new parents, we developed STORK (*S*upporting the *T*ransition to parenthood through *O*nline sexual and Relationship *K*nowledge). The STORK program attempts to address the limitations of prior intervention programs and leverage the potential of online interventions. To our knowledge, the STORK program is the first online couple-based program designed to support birthing parents and their partners in navigating perinatal sexual changes. The program employs two key strategies: (1) providing couples with psychoeducation about common perinatal sexual changes; and (2) teaching cognitive and behavioral skills to help couples manage common challenges to sexual well-being. The entire program includes five weekly modules to be completed during pregnancy (21–25 weeks) and one final module to be completed at 2-months postpartum. Throughout the program, couples receive on-demand support by posting questions via chat and have weekly check-ins with a facilitator (i.e., a clinically trained researcher serving as their contact person throughout the program) to answer questions and for additional skills coaching.

The current paper focuses on the feasibility of the introductory portion of the STORK program that included an orientation module on how to navigate the program and the first full module of psychoeducational content. Similar online programs not aimed at expecting parents (e.g., eSense [20, 21]) have been shown to be usable and feasible, thus providing a rationale for expecting similar results regarding the STORK program. This study examined the following feasibility questions: (1) To what extent is the STORK platform functional and easy to navigate? (2) How acceptable is the program content to users? (3) To what extent do users perceive the content as relevant, clear, and inclusive/representative? and (4) How reasonable do users perceive the time commitment to be? Quantitative and qualitative feedback on these questions was collected to inform refinement of the program prior to a larger pilot.

## Methods

### Module content

**Fig. 1:**
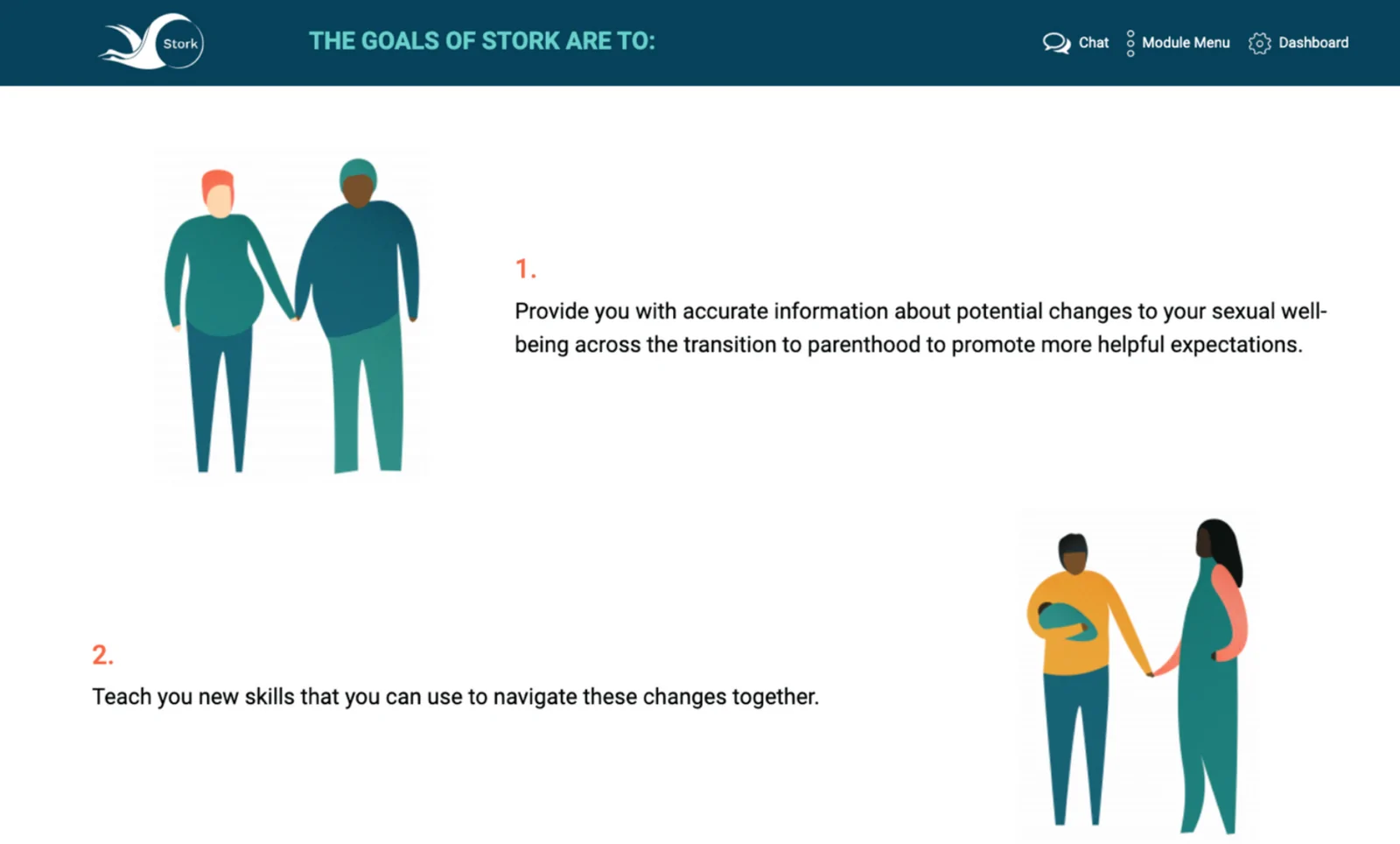
Screenshot from orientation module (i.e., module 0) outlining the goals of the STORK program

**Fig. 2:**
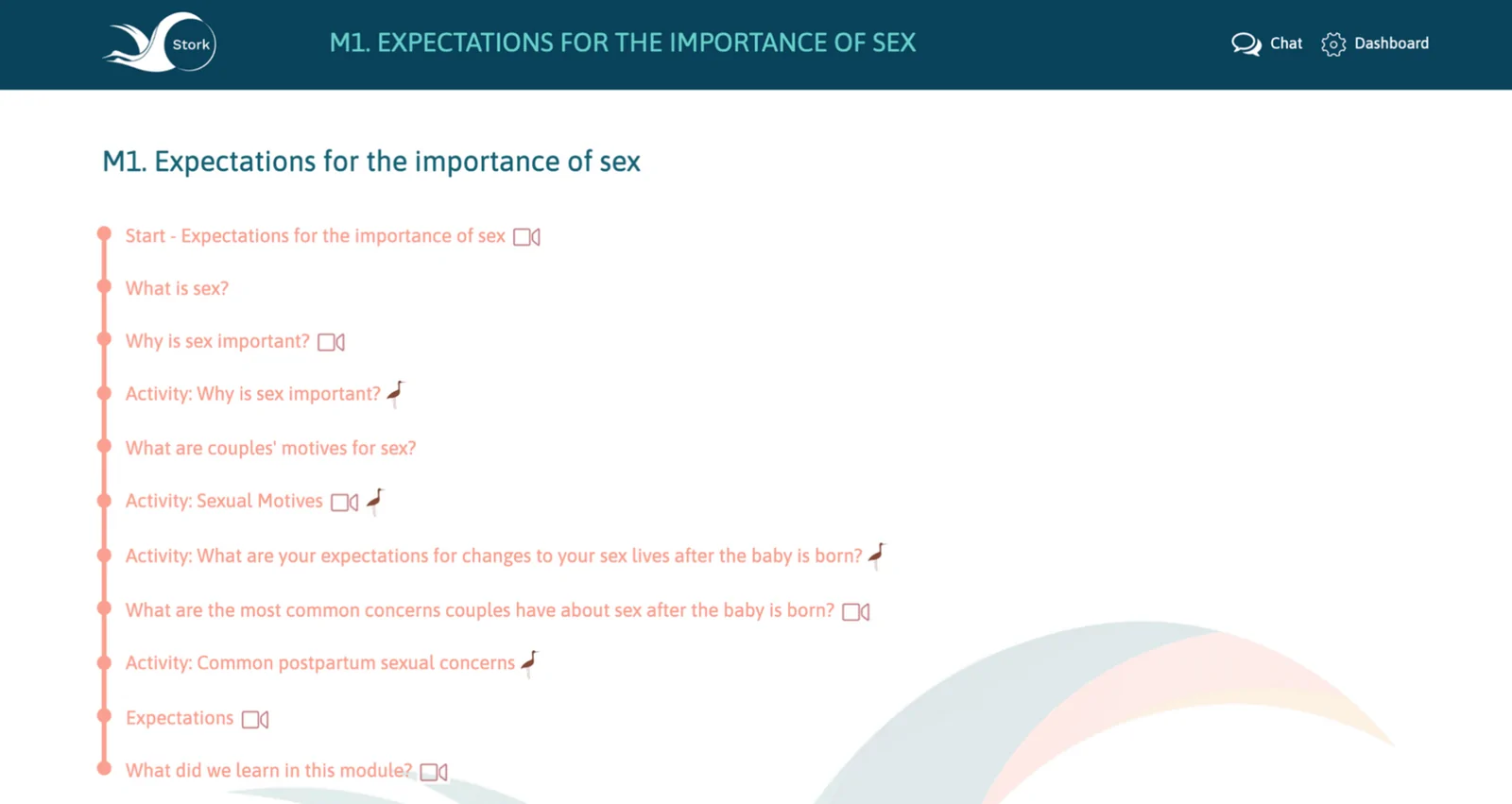
Screenshot from module 1 outlining the contents of the module

**Fig. 3:**
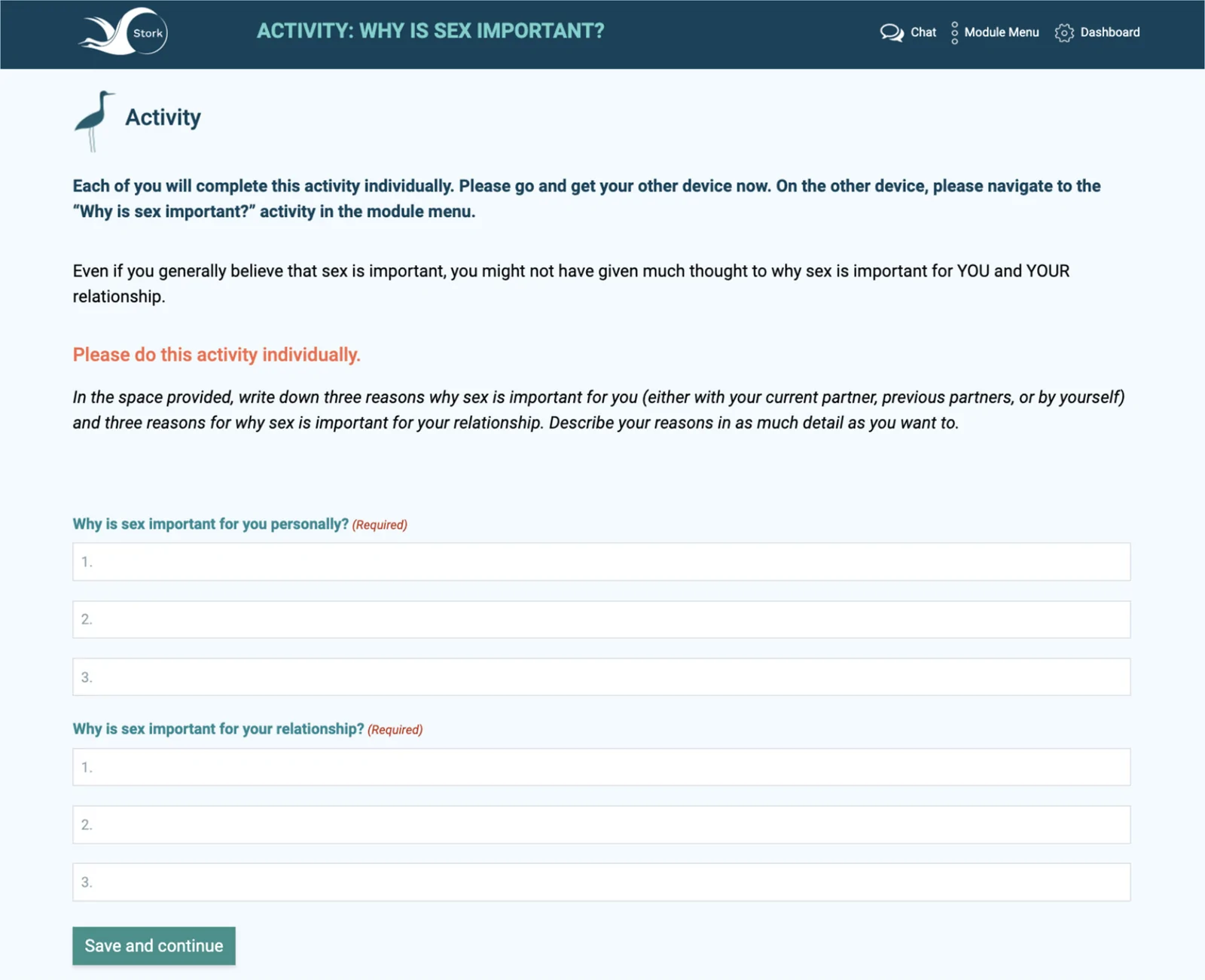
Screenshot from “Why is sex important” activity in module 1

The focus of the present study was on the orientation module and first content module of the STORK program. In developing the orientation (Module 0) and first content (Module 1) modules, we followed best practices for participant usability and engagement in online prevention programs [22, 23], including those in the transition to parenthood [18], such as short video segments (and limited text) to illustrate key concepts and interactive content that participants complete tailored to couples’ own lives (see Figs. 1, 2 and 3). Module 0 provided couples with step-by-step instructions for navigating the online platform, the goals of the program, an introduction to all team members, instructions on interacting with program facilitators (e.g., describing the chat function and weekly check-ins; not relevant for this feasibility study), and an overview of the STORK program content. Content for Module 1 and the remainder of the STORK program was informed by a growing body of dyadic longitudinal research on biopsychosocial risk and protective factors for couples’ sexual well-being across the transition to parenthood (reviewed in [1]). Module 1 included psychoeducation and interactive activities that encouraged couples to reflect on their expectations for the importance of sex for their lives and relationships, identify their unique reasons for having sex, and consider possible sexual concerns that may arise after the birth (see Figs. 1, 2 and 3 for screenshots of the STORK platform). Content in the modules was delivered through a combination of brief videos, written psychoeducational material, and interactive activities, including reflection prompts, and skills-based exercises designed to help couples apply module content to their own experiences. Because the present study evaluated only the orientation module and first psychoeducational module, the current paper describes these components in greatest detail; the full STORK program was subsequently refined based on participant feedback and later pilot testing and will be described more comprehensively in future reports.

### Participants

Participant couples were recruited across Canada and the United States of America between August and December, 2023 through partnerships with prenatal services (e.g., posters placed in ultrasound clinics, obstetrics and gynecology offices, etc.), online dissemination strategies (e.g., ads posted on the lab’s social media and shared by other accounts), and word-of-mouth. Inclusion criteria required both partners to be at least 18 years of age, speak and read English fluently, live together, and be in a relationship with each other for at least 1 year. Additionally, one partner had to be 12–25 weeks pregnant with both partner’s first child. We planned to exclude any couples who were instructed by a healthcare professional to avoid sexual activity during pregnancy. Potentially fraudulent contacts were identified using a combination of predefined screening criteria and researcher judgment. Indicators included discrepancies between IP-based and self-reported location, endorsement of fabricated recruitment sources in the initial contact survey which researchers knew to be false, formulaic or unusually familiar phrasing in communications, suspicious email addresses, and disproportionate emphasis on compensation. No participant was excluded on the basis of a single indicator; rather, determinations were made holistically based on the accumulated evidence. Of 76 potential participant couples, 36 were considered scams/fraudulent contacts (i.e., individuals who intentionally misrepresent themselves to participate in the study, as determined by inconsistent or dishonest responses to questions during the screening process). Of the 40 remaining couples, 3 declined to participate pre-enrollment, 21 did not reply to us after initially expressing interest, and 2 were lost to follow-up after some contact with us about the study. Additionally, 3 couples were ineligible after screening (one couple already had children, one couple was over 22-weeks gestation, and one couple was not fluent in English). Our study had a final sample of 11 eligible and enrolled couples (22 individuals). As this was an early feasibility study, the purpose of the sample was to provide preliminary descriptive information on feasibility metrics and to identify usability issues through qualitative feedback, rather than to power hypothesis testing. Recommendations for similar studies suggest samples of at least 8–10 participants to reach saturation of themes regarding qualitative feedback (i.e., no new problems regarding usability identified; [24]). Demographic and pregnancy characteristics can be found in Table 1.

**Table 1:** Descriptive characteristics of the sample (*N* = 11 couples, 22 individuals)

	*Pregnant partner*		*Non-pregnant partner*	
	M *(range)*	SD or %	M *(range)*	SD or %
Age (years)	32.55 (9)	2.54	33.73 (15)	3.95
Country of residence				
United States	8	72.7%	––	––
Canada	3	27.3%	––	––
Sex				
Female	11	100.0%	––	––
Male	––	––	11	100.0%
Gender^a^				
Woman	11	100.0%	––	––
Man	1	9.1%	11	100.0%
Sexual orientation				
Straight/heterosexual	10	90.9%	11	100.0%
Bisexual	1	9.1%	––	––
Culture				
White English Canadian or White American	9	81.8%	9	81.8%
White Eastern European	2	18.2%	3	27.3%
East Asian	1	9.1%	––	––
Indigenous	––	––	1	9.1%
South East Asian	––	––	1	9.1%
Education (total years of schooling)	18.6 (6)	2.3	16.9 (9)	2.5
Relationship status				
Married	7	63.6%	––	––
Common law	3	27.3%	––	––
Living together (not common law or married)	1	9.1%	––	––
Relationship duration (months)	80.9 (122)	46.8	––	––
Pregnancy weeks	19.7 (13)	3.9	––	––
Complications during current pregnancy				
No	21	95.5%	––	––
Yes, perceived early miscarriage	1	4.5%	––	––
Number of previous pregnancies				
0	12	54.5%	––	––
1	8	36.5%	––	––
2	1	4.5%	––	––
3	1	4.5%	––	––
Number of previous pregnancy losses				
0	13	59.1%	––	––
1	7	31.8%	––	––
2	2	9.1%	––	––
Difficulty of current pregnancy	3.6	2.0	––	––

In some sociodemographics, total exceeds 100% since people can report multiple responses (e.g., gender, culture)

^a^One participant selected all response options (woman, man, non-binary, agender, gender neutral, genderqueer, pangender, bigender, demigirl, demiboy, neutrois, genderfluid, genderflux)

### Measures

All measures were completed individually by each partner at the end of module completion. Feasibility was assessed using descriptive indicators of usability, acceptability, relevance, clarity, inclusivity/representativeness, and time burden; no a priori numerical feasibility thresholds were specified, as the purpose of the study was program refinement rather than formal progression testing. Selection of measures was informed by prior feasibility studies of online programs regarding sexuality (see [20]); a full description of items is provided in Supplemental.

#### Quantitative measures

##### Sociodemographics

Each participant self-reported their age, country of residence, biological sex, gender, sexual orientation, culture, total years of schooling, relationship status and duration, and pregnancy weeks.

##### Pregnancy information

Participants self-reported on prior pregnancy experiences (number of pregnancies, history of pregnancy loss) and on current pregnancy experience (experience of complications, difficulty of current pregnancy from *1—not at all difficult* to *10—extremely difficult)*.

##### Acceptability

After completing the initial STORK program’s modules, couples self-reported on acceptability of the platform using 11 researcher-derived items modeled after prior similar work [20]. Items assessed aspects such as enjoyment, overall organization, and amount of information for module 0 and module 1 using 7-point Likert-type response scales (e.g., *1—I did not enjoy it at all* to *7—I enjoyed it very much*; *1—extremely confusing* to *7—extremely clear and logical*; *1—not enough information to understand the program*, *4—perfect amount of information to understand the program*, *7—too much information or information not necessary to understand the program*, respectively). Participants also provided open-ended suggestions for improvement.

##### Content and layout

Participants self-reported on 9 researcher-derived items [20] assessing the content and layout of information presented in the STORK program such as platform layout, clarity and appropriateness of content using 7-point Likert-type response scales (e.g., *1—extremely confusing* to *7—extremely clear and logical; 1—not at all appropriate* to *7—very appropriate*). Three open-ended questions asked about areas that were covered well, areas of improvement, and confusing terms/ideas.

##### Functionality

Two researcher-derived items [20] assessed ease of navigation and the relevance of graphics (i.e., images/videos) using 7-point Likert-type response scales (*1—extremely difficult or impossible* to *10—extremely easy*; *1—not helpful at all* to *7—very helpful*); an open-ended question asked about specific suggestions to functionality of the platform.

##### Inclusivity

Participants self-reported on their perceptions regarding inclusivity of the platform using 4 researcher-derived items completed on a 7-point Likert-type response scale (e.g., *1—Extremely exclusive and/or lacks diversity* to *10—Extremely inclusive and/or diverse*). One open-ended question asked about specific suggestions to improve inclusivity of the program.

##### Time commitment

Participants self-reported how long it took them to complete the modules, and whether they considered this amount of time reasonable using a dichotomic (yes/no) item; if they responded negatively, then they were invited to provide reasons using an open-ended question.

#### Qualitative measures

After completing the module, a semi-structured interview was conducted by the first or third author. The interview included open-ended questions about the web platform, as well as specific questions regarding its content, relevancy, clarity, organization, esthetics, navigation, and functionality. The content of the previously completed open-ended survey questions was not coded in the qualitative analyses; they were rather used as a prompt to facilitate participant reflection on specific aspects of the program prior to the interview to help them identify key points of interest and better articulate their experiences during the qualitative interview.

### Procedure

Potential participants were scheduled for a 10-min phone screen with a research assistant to determine eligibility. Although it was suggested that both members of the couple attend the phone screen, we only required speaking with one partner. In cases where one partner completed the call, we followed up with the other partner via email to provide them with information about the study and confirm their interest in participating. Couples were only enrolled after both partners confirmed their interest in participating. Upon enrollment, both partners were individually sent a link to an online survey including the consent form and pre-program measures (i.e., sociodemographics) via Qualtrics (https://www.qualtrics.com) and subsequently were booked for a 2-h session with a researcher. During this session, couples were asked to complete the orientation and first modules of the STORK program while a research assistant was available to support them with their platform navigation experience (i.e., the researcher was present during the whole session and available to provide support should couples have any questions/feedback). After completing the modules, couples were asked to individually complete a short (10–15 min) online survey evaluating the program modules and, finally, to participate in a semi-structured interview where they were able to expand on their comments about the feasibility of the platform/content. Couples were compensated with $80 CAD or USD equivalent per couple and were granted free access to the full STORK program upon completion. This study was approved by the [*masked*] Research Ethics Board [*ref masked*].

### Analyses

Mean scores (with standard deviations) were computed for feasibility indices. Qualitative responses were coded with thematic analysis using a deductive approach and following Marks’ team-based thematic analysis [25] and a guide of this method provided by Allsop and colleagues [26]. First, two coders (first and fourth author) independently conducted open coding for each response, assigning a brief conceptual “code” to capture the essence of what the participant was discussing [25]. After completing the open coding, open codes were consolidated into core themes through team discussion. The coders met to compare their initial codes, identify any discrepancies or missed codes, and resolve differences through discussion and consensus. Throughout the coding process, the coders maintained analytic memos documenting coding decisions, disagreements, emerging interpretations, and rationale for refinement of themes, which were discussed during team meetings. The coders met with one of the project’s team leads who had not participated in the coding process (i.e., a non-coder; last author). Following the recommendation by Allsop and colleagues [26], the inclusion of a non-coder in discussions of codes and themes ensured diverse perspectives while staying aligned with the study’s goals. Together, the coders and the non-coder worked to refine the themes by merging conceptually overlapping codes, removing codes that lacked sufficient support across responses. Clear definitions for the core themes were established and agreed upon by both coders.

## Results

### Quantitative results

#### Acceptability

For module 0, participants rated it as “clear and logical” (*M* = 5.6, *SD* = 1.3), reported its completion as “enjoyable” (*M* = 4.7, *SD* = 1.8), the organization of its content as “very clear and logical” (*M* = 5.8, *SD* = 1.4), and the amount of information presented in this module as the “perfect amount of information to understand the program” (*M* = 4.6, *SD* = 1.1; the scale for this item ranged from 1 = “not enough information to understand the program”, to 4 = “perfect amount of information to understand the program”, to 7 = “too much information or information not necessary to understand the program”). Overall, participants rated the content of module 0 as “helpful” (*M* = 5.1, *SD* = 1.4).

For module 1, participants rated this module in general as “clear and logical” (*M* = 5.9, *SD* = 1.2), reported its completion as “very enjoyable” (*M* = 6.1, *SD* = 1.3), the organization of its content as “extremely clear and logical” (*M* = 6.2, *SD* = 1.0), and the amount of information presented as the “perfect amount of information to understand the program” (*M* = 4.1, *SD* = 0.6). Overall, participants rated the content of module 1 as “very helpful” (*M* = 5.7, *SD* = 1.6) and considered the content of this module as “very relevant” to their lives (*M* = 6.1, *SD* = 1.0).

#### Content and layout

Participants rated the layout of the STORK platforms as “clear and logical” (*M* = 5.3, *SD* = 1.2), the overall clarity of the content as “very clear” (*M* = 5.8, *SD* = 1.3), the overall platform as “very attractive” (*M* = 5.6, *SD* = 1.1), and the look of the platform as “very appropriate” to the subject matter (*M* = 6.2, *SD* = 1.2).

#### Functionality

Participants considered the web platform to be “very easy to navigate” (*M* = 5.6, *SD* = 1.1) and the videos, with and without accompanying graphics (i.e., animations) were found to be “very helpful” (*M* = 6.0, *SD* = 1.1).

#### Inclusivity

Participants rated the program to be “extremely inclusive” (*M* = 6.0, *SD* = 1.4) by considering and representing the experiences of many different people (such as in relation to race, gender, sex, ability, socioeconomic status, age etc.). This was also the case for the graphics and illustrations (*M* = 6.1, *SD* = 1.2), the language (*M* = 6.0, *SD* = 1.5), and the content (*M* = 6.1, *SD* = 1.6).

#### Time commitment

Most (95.5%) participants considered that the time commitment required by the STORK program was reasonable. Ninety-one percent of participants reported that it would be reasonable to expect them to complete a module with their partner once per week for 5 weeks in pregnancy and a final module at 2-months postpartum. Of those who responded negatively to this question and provided an open-ended response (*n* = 2), reasons included considering that it would be harder to commit in the postpartum period and that “maybe it would go faster now that I’m used to the browser navigation but I’m a very busy person and this is just extracurricular to me”.

### Qualitative responses

Thematic analysis of participants’ responses revealed four primary themes: (1) usability and accessibility, (2) content quality, (3) inclusivity and representation, and (4) impact and engagement. Within these overarching themes, codes emerged that highlight specific aspects of participant experiences. Definitions and examples of each of these themes are presented below. Total frequency counts for each theme/code are presented in Supplemental.

#### Usability and accessibility

This theme focuses on participants’ experiences with the platform’s design, navigation, and functionality, emphasizing user-friendliness and accessibility. Participants consistently described the platform as user-friendly. Many users highlighted the clarity and simplicity of the layout, which contributed to an accessible and engaging experience. *Navigation and Interface*. Participants unanimously reported that the platform’s navigation was straightforward and intuitive. The design was described as “clear” and “easy to navigate.” One participant commented, “I think if you’ve ever done an online course or something like that, it’d be pretty easy to follow.” *Visual Appeal.* Participants appreciated the color scheme, balanced layout, and typography: “It’s colourful, easy to read, even the text,” and “Good font, background, like all I thought visually it was really well put together”. The platform was described as “pretty” and “pleasing,” with one participant highlighting that it “feels very welcoming.” *Amount and Length of Content.* Participants described the balance of content types—videos, images, and text—to be well-proportioned and digestible: “It felt like there was a pretty good balance between videos, images and graphics, and then activities”. A key noted strength was the ability to choose between reading and watching video content (or both), allowing flexibility in engagement: “It’s good having both; if you can’t connect to a video or don’t feel like reading, you have the choice to do either.” *Functional Suggestions.* Some participants suggested additional features to enhance usability (e.g., a progress bar, accessibility features like closed captions and color-blind-friendly contrast settings): “I wonder if it’s even possible to have a progress bar?” and “I think closed captioning on the videos would be more accessible,” One participant also noted, “Maybe having something in there about making sure to save your responses”.

#### Content quality

This theme captures perceptions of the content’s relevance, depth, and clarity, as well as how it engaged participants. The content was largely perceived as relevant, engaging, and comprehensive, with opportunities for enhancement in specific areas. Participants appreciated the logical progression and interactive elements but noted areas where content could be expanded for deeper understanding. *Relevance.* Participants described the content as meaningful and engaging, for example: “I think the information was super relevant and I think we liked it”. Many remarked that it often introduced new insights beyond common knowledge: “This is new information and not just common sense”. *Comprehensiveness.* Overall, participants found the content thorough, with one noting “nothing important was left out” and another remarking “I felt it was really thorough and very informative”. Although some suggested additional information on topics such as post-birth expectations and effective communication strategies: “I did notice it was a little hard for us to answer some of the questions because we’ve never been parents before, so we don’t really know how we’ll feel after”. *Understanding and Engagement.* Participants valued the logical flow of the content and the use of videos to provide a more personal element to the information. One participant remarked, “It went through the explanation of sex, why people do it, and what it would be like post-baby. It had a good flow and made sense to us.” Another commented “I felt pretty engaged while going through it. I think the questions were good to reflect [on]”. *Activities.* Interactive exercises were well-received, with participants highlighting their utility in prompting discussions and reflections and provided relatable peer insights: “I liked that it gave you an opportunity to come up with your own answers but also showed you other potential responses, which made me think, ‘Oh, I didn’t consider that, but it definitely applies to me’”.

#### Inclusivity and representation

This theme examines the platform’s inclusivity in content, design, and representation of diverse experiences. The platform was praised for its inclusive approach, particularly in language and imagery. Participants appreciated the platform’s efforts to use gender-neutral language and depict diverse experiences. One participant commented: “You can’t tell the gender of the people, which is good.” Another noted, “I really like the inclusive language and the huge effort to be inclusive.” Suggestions for improvement included the inclusion of scenarios relevant to couples who used medically assisted reproduction.

#### Impact and engagement

This theme explores how the platform affected participants’ emotional and relational experiences while completing it. The platform facilitated meaningful conversations and reflections, fostering emotional connection and engagement between partners. Participants reported increased discussions with their partners and a desire to continue the program. *Reflection, Connection, and Communication*. Participants reported that the content in the STORK program’s modules encouraged open conversations about potential relationship changes, helping participants feel understood and validated: “Even some of the wording and things to consider that maybe I hadn’t thought about made me reflect and opened up conversations with my partner.” Participants also appreciated opportunities for greater relatedness with their partner: “I really like the interactive side of things, like getting to learn my partner’s perspective” and “I know [having sex] is important for our relationship and the well-being of our relationship, but…it was nice to have that opportunity to sit down and talk about it”. *Interest in Continuing the Program.* Many participants expressed enthusiasm for engaging with future modules, finding the program valuable and thought-provoking. As one participant stated: “I definitely would 100% consider doing the whole program because I think it’s super valuable.” Another shared: “I’m curious to see the other modules.”

## Discussion

The purpose of the current study was to assess the feasibility of the orientation and introductory modules of the STORK program: the first online couple-based program to support new parent couples’ perinatal sexual well-being. Participants reported high satisfaction with the program’s usability, organization, and inclusivity and rated the content as clear and highly relevant. Suggested changes to the content were minimal. Participants reported that the program prompted meaningful reflection and communication between partners and most found the time commitment reasonable and expressed interest in completing the full program. Thus, the pilot module was found to be highly feasible. These findings are important given that perinatal sexual health remains an under-addressed area in both research and routine prenatal care, despite evidence that sexual changes are common and often distressing for couples during the transition to parenthood [27, 28].

The findings highlight several strengths of the STORK program. Participants consistently referred to the platform’s user-friendly design, describing it as intuitive, easy to navigate, and visually appealing. The program’s content was also well-received, with participants finding it clear, logically structured, and relevant to their experiences. Importantly, the interactive elements effectively engaged participants, fostering meaningful self-reflection and communication between partners about sexual topics, which are often difficult to address during the perinatal period [9]. This result is notable because difficulties in talking openly about sexual concerns may contribute to misunderstandings, distress, or unmet expectations during the perinatal period; as such, an intervention that normalizes these conversations may address an important relational pathway linked to sexual well-being. Participants also highlighted the program’s commitment to inclusivity. Participants provided some suggestions to the content of the module and most of these suggestions for additional information (e.g., information about effective communication, more information on expectations for sex after childbirth) are, in fact, covered in the subsequent modules of the program. This pattern of feedback also suggests that participants were not only passively satisfied with the content but were actively engaging with it in ways that may support refinement and future implementation. The consistent positive feedback, along with minimal suggestions for content changes, underscores the feasibility of the STORK program in supporting perinatal sexual well-being. More broadly, these findings support the potential value of digital, couple-based formats for delivering sexual health education in a way that is private, accessible, and easy to integrate into prenatal preparation.

Given high levels of engagement and positive participant experiences, this program has strong potential for implementation in clinical and community settings to supplement traditional perinatal education, supporting past research on perinatal sexuality programs [17, 29]. In particular, the online format may help address barriers that commonly limit access to sexual health support, such as time constraints, discomfort discussing sexuality in person, and limited availability of providers with training in perinatal sexual well-being. The current data do not allow us to assess retention or efficacy for the full program. Future work should include a full pilot of the complete intervention, including all modules, to evaluate implementation considerations such as cost, training requirements, and scalability. This should be followed by a pilot randomized controlled trial to assess participant retention, preliminary estimates of effect, and, subsequently, the long-term efficacy of the full STORK program. Additionally, comparative studies against standard prenatal care or other interventions would help assess efficacy. Expanding the program’s scope to include tailored content for diverse populations, such as LGBTQ+ couples and those using medically assisted reproduction, could further enhance its accessibility and impact.

While the study provides encouraging preliminary findings, some limitations should be noted. For example, although the presence of a researcher to offer support, answer questions, and help identify potential navigation or platform issues has been found to be beneficial in prior studies [17], it may have made the experience more structured than real-world use, potentially increasing participant engagement and contributing to socially desirable qualitative feedback. Another limitation relates to the characteristics of the sample. Our focus on first-time parents was due to these couples’ greatest need for guidance during the transition to parenthood but limits generalizability. The program may also be relevant for multiparous couples; testing this applicability might be the focus of subsequent pilot testing. Moreover, some of the positive outcomes reported may be influenced by participants’ motivation and willingness to take part in such a study, as well as by social desirability bias given the sensitive nature of the topic and the presence of a researcher. The sample was highly educated (with most participants having completed some postsecondary education), white, and heterosexual, which may limit the generalizability of findings to individuals from more diverse socioeconomic backgrounds. Although participants rated the program as inclusive, the relative homogeneity of the sample limits our ability to assess whether the program is experienced as inclusive across diverse populations. An evaluation of the program in a more diverse sexual and gender/sex sample has been conducted in parallel work [citation masked for review]. Finally, because the study focused on prenatal participation, future research should explore the program’s usability and efficacy postpartum to assess retention and long-term benefits.

## Conclusions

The findings from this study provide preliminary support for the feasibility of the initial modules of the STORK program as an online intervention designed to help couples navigate perinatal sexual changes. Participants responded positively to the platform, reporting that it was user-friendly, engaging, and relevant to their experiences. Both quantitative ratings and qualitative feedback emphasized the clarity and inclusivity of the content, as well as the program’s ability to stimulate meaningful conversations between partners. The highly positive participant feedback suggests that the STORK program may be a promising resource to address a gap in perinatal sexual education by offering structured, accessible, and evidence-based support. Moreover, by delivering an intervention that does not require a highly trained professional for delivery, the STORK program might represent a potentially cost-effective and scalable approach to supporting perinatal sexual well-being. By providing couples with the knowledge and tools to navigate sexual changes, programs like the STORK program can play an important role in promoting sexual well-being during the transition to parenthood.

## Supplementary Information

Supplementary Material 1.

## Data Availability

Data are available upon request.
