# Supplementary material for: Supporting couples’ perinatal sexual well-being: a feasibility study of the introductory modules of a novel prevention program (STORK)
Source: Pilot Feasibility Stud. 2026 Jul 21;12:150. doi: 10.1186/s40814-026-01878-8 (PMC13670602; doi:10.1186/s40814-026-01878-8)
Supplement: Supplementary file 1 [file 40814_2026_1878_MOESM1_ESM.docx]

**Supplemental Materials**

**Measures**

***Quantitative Measures***

The wording of some of the questions in this survey is not perfect and may not reflect your lived experience and/or identity. Our research team continues to learn as we examine and reflect on our own practices to develop more inclusive measures. There will be an opportunity at the end of this survey to provide feedback.

*Please answer in a manner that you feel best reflects your experience and feel free to contact us if you have any questions or concerns.*

1. How did you find out about this study?

- Instagram
- Facebook
- Twitter
- Poster/flyer
- Newspaper
- Community group
- Other (please specify) [text box]

2. What is your age in years? _____.

3. What state/province/territory do you currently live in?

- Drop down list of states/provinces/territories

4. What is your place of birth?

- Canada
- United States
- Western Europe
- Eastern Europe
- Africa
- Asia
- Australia
- Middle East
- Latin America / South America
- Caribbean
- Other (specify if you wish) [text box]

5. Which of the following reflect your ethno-racial background? (Please check all that apply)

1.  White French Canadian/Québécois

2.  White English Canadian or White American

3.  White Western European (e.g. England, Sweden)

4.  White Eastern European (e.g. Hungary, Russia)

5.  Indigenous (First Nations, Métis or Inuit)

6.  Black Canadian

7.  Black African (e.g. Ghana, Kenya, Somalia)

8.  North African (e.g. Algeria, Morocco, Tunisia)

9.  Black Caribbean (e.g. Haiti, Jamaica)

10. Middle Eastern (e.g. Egypt, Iran, Israel, Saudi Arabia)

11. Latin American (e.g. Argentina, Mexico, Nicaragua)

12. East Asian (e.g. China, Japan, Korea, Taiwan)

13. South Asian (e.g. India, Sri Lanka, Pakistan)

14. South East Asian (e.g. Vietnam, Malaysia, Philippines)

15. Indo-Caribbean (e.g. Guyanese with origins in India)

16. Something else, please specify __________ [text box]

6. Are you perceived or treated as a person of colour?

Y/N

7. (if selected Indigenous) Do you identify as Two-Spirit?

1. Yes
2. No

8. How many years of schooling do you have (starting from first grade)?

[Dropdown menu ranging from 1 to 30+]

9. Which of the following best describes your current employment status? (Please check all that apply.)

- Employed outside of the home (full-time)
- Employed outside of the home (part-time)
- Employed inside of the home (full-time)
- Employed inside of the home (part-time)
- Paid parental leave (full-time)
- Paid parental leave (part-time)
- Unpaid parental leave (full-time)
- Unpaid parental leave (part-time)
- Student (full-time)
- Student (part-time)
- Unemployed
- Retired
- Unable to work due to disability
- Not listed (please specify) [text box]

10. What is the approximate total annual income of your household? (i.e., the total income for you and any partner you live with before taxes)

- $0	-	$9,999
- $10,000 - $19,999
- $20,000 - $29,999
- $30,000 - $39,999
- $40,000 - $49,999
- $50,000 - $59,999
- $60,000 - $69,999
- $70,000 - $79,999
- $80,000 - $89,999
- $90,000 - $99,999
- $100,000 and over

11. When we describe the gender identity of the participants in our study, which of these categories would you like us to include you in? (Please select all that apply)

- Man
- Woman
- Non-binary
- Agender
- Gender neutral
- Genderqueer
- Pangender
- Bigender
- Demigirl
- Demiboy
- Neutrois
- Genderfluid
- Genderflux
- Something else/prefer to self-describe (please specify if you wish) ____________
- Prefer not to say

12. Sometimes we need to group together gender categories to ensure they are large enough to conduct statistical analyses. Which of these groups would you like us to include you in, if at all? (Please select one)

- Man
- Woman
- Non-binary (e.g., genderfluid, genderqueer)
- I would not like to be included in these types of analyses

13. What **sex were you assigned at birth**? This means the sex that someone – typically a medical professional – gave you when you were born, which is often noted on your birth certificate.

- Male
- Female
- Something else (open-ended)
- Prefer not to say

14. Were you born with a variation in your physical sex characteristics? This is sometimes called being intersex, or having a difference, divergence, diversity or variation in sex development (DSD or VSC).

- Yes
- No
- Unsure
- Prefer not to say

15. When we describe who participated in our study, which of these categories would you like us to include you in? (Please select one)

- A cisgender category (refers to people who are the same gender and/or sex that they were assigned at birth)
- A trans/transgender category (usually refers to people who were given a gender or sex label at birth that does not accurately represent them today)
- Neither cisgender nor transgender describe me because (please specify):
- Unsure because (please specify):

16. And which of these categories would you like us to include you in? (Please select one)

- Binary (someone who identifies as exclusively a man/male or woman/female)
- Nonbinary (someone who has an identity other than exclusively woman/female or man/male)
- Neither binary nor nonbinary describe me because (please specify):
- Unsure because (please specify):

17. People describe their sexual orientation in different ways. Which expression best describes your current sexual orientation? If no expression describes yourself, check “Not listed” and write the answer that describes you personally.

- Bisexual
- Asexual
- Gay
- Lesbian
- Straight/Heterosexual
- Pansexual
- Queer
- I have not figured out my sexual orientation or am in the process of figuring it out (Questioning)
- Not listed (specify if you wish) [text box]

18. Which of the following best describes your relationship with your romantic partner?

- Living together (not common-law or married)
- Common-law
- Married
- Not listed (specify if you wish) [text box]

19. How long have you been in a relationship with your partner? Please count from the start of the relationship. For example, if you dated for 2 years and have been married for 2 years and 3 months you should answer 4 years and 3 months.

______ Years &_______Months

20. Are you or your partner currently pregnant?

Y/N

16.1. If so, how many weeks pregnant? _______.

21. Have you or your partner had any complications during your pregnancy?

Y/N

17.1. If so, which? ______________.

23. How many times have you been pregnant before your current pregnancy?

____ times

24. Have you experienced a previous pregnancy loss?

Y/N

If yes, please specify how many times this has happened to you: [text box]

25. On a scale from 1 (not at all difficult) to 10 (extremely difficult), how would you describe your pregnancy experience to date?

*Consider module 0 (the orientation module). Overall, how would you rate your impressions of this module?*

1 – extremely confusing; 7 – extremely clear and logical

*Did you enjoy the experience of working through module 0?*

1 – I did not enjoy it at all; 7 – I enjoyed it very much

*How would you rate the amount of information presented to you in this module?*

1 – not enough information to understand the program; 4 – perfect amount of information to understand the program; 7 – too much information or information not necessary to understand the program

*Overall, how helpful was the content of this module?*

1 – extremely unhelpful; 7 – extremely helpful

*What areas do you think could use improvement? Do you have any specific suggestions for changes to make to the orientation module?*

______________________________.

*Now consider Module 1. Overall, how would you rate your impressions of this module?*

1 – extremely confusing; 7 – extremely clear and logical

*Did you enjoy the experience of working through module 1?*

1 – I did not enjoy it at all; 7 – I enjoyed it very much

*How would you rate the amount of information presented to you in this module?*

1 – not enough information to understand the program; 4 – perfect amount of information to understand the program; 7 – too much information or information not necessary to understand the program

*Overall, how helpful was the content of the module?*

1 – extremely unhelpful; 7 – extremely helpful

*Overall, how relevant was the content in this module to your life?*

1 – not all relevant, 7 – extremely relevant

*What areas do you think could use improvement? Do you have any specific suggestions for changes to make to module 1?*

______________________________.

*How would you rate the overall organization of the content in module 0?*
1 – extremely confusing; 7 – extremely clear and logical

*How would you rate the overall organization of the content in module 1?*
1 – extremely confusing; 7 – extremely clear and logical

*How would you rate the layout of the STORK platform content pages?*

1 – extremely confusing; 7 – extremely clear and logical

*How would you rate the overall clarity of the content?*

1 – extremely confusing; 7 – extremely clear

*How attractive was the STORK web platform overall?*

1 – not at all attractive; 7 – very attractive

*How appropriate was the look of the STORK web platform to the subject matter?*

1 – not at all appropriate; 7 – very appropriate

*What areas do you think were covered well?*

__________________________.

*What areas do you think could use improvement? Do you have any specific suggestions for changes to make the content clearer or more engaging?*

__________________________.

*Were there any specific terms or ideas that were particularly confusing?*

__________________________.

*How would you rate the ease of navigating the STORK web platform?*
1 – extremely difficult or impossible; 10 – extremely easy

*There were videos, with and without accompanying graphics (i.e., animations) in the modules. How helpful did you find the graphics?*

1 – not helpful at all; 7 – very helpful

*What, if anything, would you improve about the STORK web platform? How might you change the layout or organization of the STORK web platform to make it easier to navigate?*

*­­*__________________________.

*Overall, how would you rate the inclusivity and diversity of the STORK program? In in other words, to what extent did you find that the STORK content considered and represented the experiences of many different people (such as in relation to race, gender, sex, ability, socioeconomical status, age etc.)?*

*1 – Extremely exclusive and/or lacks diversity; 10 – Extremely inclusive and/or diverse*

*How inclusive and diverse were the graphics and illustrations in these modules?*

*1 – Extremely exclusive and/or lacks diversity; 10 – Extremely inclusive and/or diverse*

*How inclusive was the language used in these modules (for example by considering different levels of education, different gender identities, etc.)?*

*1 – Extremely exclusive; 10 – Extremely inclusive*

*How relatable was the content presented in these modules for you?*

*1 – Extremely unrelatable; 10 – Extremely relatable*

*What, if anything, could be improved to make the STORK program more inclusive and diverse? More relatable?*

*__________________________.*

*When working through the content, how long did it take you and your partner to complete module 0?*

__________________________.

*When working through the content, how long did it take you and your partner to complete module 1?*

__________________________.

*Was this a reasonable amount of time you could commit for once a week (across 5 weeks in pregnancy, and 1 week around 2 months postpartum)?*
*Y, N*

*Do you think it would be reasonable to expect you to do this program together with your partner across 5 weeks in pregnancy, and 1 week around 2 months postpartum?*

*Y, N*

*If no, why not: _________________.*

*What were your overall impressions of the modules you just completed?*

_________________________________________.

*Is there anything else that you would like the researchers to know about your experience?*

__________________________.

***Interview Questions***

*You reported on some of the aspects we will ask you about in your questionnaire, but we wanted to give you an opportunity to expand in more detail on any areas that could be important to improve the STORK program.*

*After reviewing the Intro module (M0), was there anything you felt you needed more information about before beginning STORK? For example, in terms of how to navigate the platform?*

*What were your overall impressions of module 1 (e.g., the proposed content)? Did anything stand out, good or bad?*

*(if bad, what do you think could have made that experience better? What would you have preferred?)*

*What did you think of the flow of information? The amount of information?*

*What do you think was covered well? What could use improvement?*

*Were you able to understand the terms and topics presented to you?*

*Did the organization of the website make sense? Is there anything you would change?*

*What were your thoughts on the layout of the page? Is there anything you would change (e.g., the balance of videos and text)?*

*What did you think of the way content was presented (some content was presented as text, video, or activity)? Was there enough variety? Was there anything you think would be better as a different delivery method?*

*What did you think of the activities in the module? Were they relevant? How easy vs difficult were they for you?*

*In Module 1, you were asked to complete four activities. Consider each of these activities while answering the following questions.*

*In the activity about reasons why sex is important for you…*

*How clear were the instructions for this activity?*

*Was the amount of information presented to you in this activity sufficient for you to complete the activity?*

*Do you think the way the activity was presented was reasonable to you (complete individually and share with your partner at the end)?*

*What aspects of the activity (instructions, completion individually or as a couple) could use improvement? Do you have any specific suggestions for changes to make the activities clearer or more engaging?*

__________________________.

*In the sexual motives card sort activity…*

*How clear were the instructions for this activity?*

*Was the amount of information presented to you in this activity sufficient for you to complete the activity?*

*Do you think the way the activity was presented was reasonable to you (complete individually and share with your partner at the end)?*

*Did you like the way this was presented to you? (Would you have preferred this to be an instructional video instead of text?)*

*What aspects of the activity (instructions, completion individually or as a couple) could use improvement? Do you have any specific suggestions for changes to make the activities clearer or more engaging?*

__________________________.

*In the activity about expected sexual changes at postpartum…*

*How clear were the instructions for this activity?*

*Was the amount of information presented to you in this activity sufficient for you to complete the activity?*

*Do you think the way the activity was presented was reasonable to you (complete individually and share with your partner at the end)?*

*What aspects of the activity (instructions, completion individually or as a couple) could use improvement? Do you have any specific suggestions for changes to make the activities clearer or more engaging?*

__________________________.

*In the activity about your 3 most important postpartum sexual concerns…*

*How clear were the instructions for this activity?*

*Was the amount of information presented to you in this activity sufficient for you to complete the activity?*

*Do you think the way the activity was presented was reasonable to you (complete individually and share with your partner at the end)?*

*What aspects of the activity (instructions, completion individually or as a couple) could use improvement? Do you have any specific suggestions for changes to make the activities clearer or more engaging?*

__________________________.

*What were your thoughts on the content (text, illustrations) of this module in terms of being inclusive and diverse? In other words, to what extent did you find that the STORK content considered and represented the experiences of many different people (such as in relation to race, gender, sex, ability, socioeconomical status, age etc.)?*

*Do you have suggestions for how we could improve the STORK platform to be more inclusive and diverse? More relatable?*

*Was it easy to navigate through the sections? Did you experience any difficulties? (Was it intuitive?)*

*Did you like the look of the module? What specifically did you like or not like?*

*Regarding the pictures and videos in the program, do you think they enhanced the program? Were they helpful for your understanding of the content? Did they help to maintain your attention as you worked throughout the module?*

*What would you improve about the website?*

*Is there anything else you’d like to tell me about your experience with STORK?*

**Data Analysis**

***Main Themes and Codes***

**Theme 1: Usability and Accessibility**

- **Code 1.1: Navigation and Interface (100%)**

Description: This code highlights the ease and intuitiveness of navigating the webpage, emphasizing its user-friendly design and logical structure.

- Concise, clear, easy to navigate. (“It was pretty clear and easy to navigate”)
- Navigation was intuitive.
- “I think [as] younger participants... we can intuitively figure out where things are, where the chat is, how a dashboard works in a menu.”
- **Code 1.2: Visual Appeal (100%)**

Description: This code pertains to the webpage’s inviting visual design, including a harmonious color scheme, clean typography, and balanced layout that enhance user engagement.

- Colour scheme was good, light background was good and clear font.
- “Pretty, pleasing; whiter spaces, not heavy pages.”
- “It feels very welcoming.”
- “I think it's super beautiful. I like the graphics of people.”
- **Code 1.3: Amount and length of content (100%)**

Description: Describes the balance of content types—videos, images, and text—ensuring the information is digestible, diverse, and engaging.

- Digestible
- “Good balance between videos, images, and graphics.”
- “I think it's good having both that if you can't connect to a video, you know, or you don't feel like reading you have the choice to do either.”
- **Code 1.4: Functional Suggestions (86%)**

Description: Includes suggestions to improve website functionality, such as implementing a progress bar, auto-save, or accessibility features like closed captions and colorblind-friendly contrast.

- Progress bar and table of contents
- Auto save option (“If the ‘next’ [button] is an automatic ‘save’ button…that could be it”)
- Accessibility suggestions (e.g., colour-blind friendly contrast, closed captioning on videos, spell check for text entry)
- “The only thing I can think of is the color contrast might not be necessarily colorblind friendly.”

**Theme 2: Content Quality**

- **Code 2.1: Relevance (71%)**

Description: Addresses the perceived relevance and novelty of the content, which participants found meaningful and engaging, often balancing new information with relatable reflection.

- Information was considered relevant.
- “This is new information and not just common sense.”
- Good balance between content and reflection; felt engaged.
- “We’re both learning something. Our situations are different, but the act is the same for both of us.”
- **Code 2.2: Comprehensiveness (100%)**

Description: Reflects participants’ impressions of the content’s thoroughness, noting that most key topics were covered, with some areas, like post-birth expectations, suggested for expansion.

- Nothing important was left out.
- Suggestions: Information about effective communication; expectations after birth should be expanded.
- “I felt it was really thorough and very informative.”
- “It was more I wanted to continue to work on it than that there was information that was missing.” (*it* referring to the other modules)
- “It would have been really interesting to see like even just snippets or quotes by people who have been first-time parents and like how it actually did change their sex life.”
- **Code 2.3: Understanding and Engagement (100%)**

Description: Focuses on the content’s clarity and logical flow, with videos adding a personal, relatable element. Suggestions included elaborating on certain topics for greater depth.

- Liked the progression of the module.
- “It went through the explanation of sex, like why people do it or why we do it....what would it be like now versus post-baby. It's a good flow and it made sense to us.”
- Some topics could be further clarified/elaborated on
- Videos provided a personal element
- “I found it easier…to have a person you can watch that’s sharing the knowledge”
- **Code 2.4: Activities (100%)**

Description: Describes the clarity, utility, and interactivity of activities, with participants appreciating opportunities for reflection and relatable peer examples.

- Activities: Clear, easy to follow, with sufficient information.
- Utility of activities notes
- “I feel like we had really good discussions and then we were like, oh, we have to fill something in this box to continue to the next page.”
- Comments and suggestions related to the activities
- “I liked how it like it gave you an opportunity to come up with your own answers but then it also showed you like other people’s common answers because then I was like, oh, I didn't think of that, that’s definitely something that I relate to.”

**Theme 3: Inclusivity and Representation (100%)**

Description: This code reflects the participants’ appreciation for the program’s inclusivity in design, language, and content. It highlights the intentional efforts to ensure diverse and equitable representation across gender, ethnicity, and experiences, fostering a welcoming and relatable environment. Suggestions for further improvement included incorporating scenarios for IVF couples and maintaining gender-neutral references.

- Liked the inclusive language.
- Content was described as inclusive/diverse.
- Example: “You can't tell the gender of the people, which is good.”
- “I really like the inclusive language and the huge effort to be inclusive.”
- “Liked the variety of colours and genders.”
- Suggested addition: Transition to parenthood for IVF couples.
- “I think I heard the baby, like she used a gendered pronoun and I don't know if it’s a possibility just to change it to a non-gendered pronoun for people that are like transgender or non-binary and watching it...Or for people who haven’t found out the sex of the baby.”

**Theme 4: Impact and Engagement**

- **Code 4.1: Reflection, Connection, and Communication (100%)**

Description: This code captures how the program encouraged meaningful conversations between partners while fostering emotional connection and mutual understanding. Participants appreciated relatable examples and felt their experiences were normalized, enabling them to reflect on anticipated relationship changes and discuss them openly.

- Prompted to communicate about sexual changes with each other.
- Wanting to have more conversations with their partner.
- Anticipated changes to the relationship.
- “Good conversation starter.”
- “Even some of the wordage and things to consider that maybe I hadn’t thought about. Even [moving] forward, that would maybe get your brain thinking and maybe open up a conversation with my partner at home, like what do you think about this, or have you thought about this?”
- Comfort discussing things with their partner.
- “Felt the content of the modules normalized what we were feeling.”
- “Liked the relatable examples.”
- “I really like the interactive side of things, like getting to learn my partner’s perspective.”
- **Code 4.3: Interest in Continuing the Program (71%)**

Description: Describes participants’ enthusiasm for continuing the program, emphasizing its perceived value and curiosity about upcoming modules.

- Willing and interested in using the program.
- “I definitely would 100% consider doing the whole program, ‘cause I think it’s super valuable.”
- “I’m curious to see the other modules.”
